# Study design approaches for future active-controlled HIV prevention trials

**DOI:** 10.1515/scid-2023-0002

**Published:** 2024-01-22

**Authors:** Deborah Donnell, Sheila Kansiime, David V. Glidden, Alex Luedtke, Peter B. Gilbert, Fei Gao, Holly Janes

**Affiliations:** Fred Hutchinson Cancer Center, Seattle, WA, USA; University of Washington, Seattle, WA, USA; Medical Research Council/Uganda Virus Research Council and London School of Hygiene and Tropical Medicine, Uganda Research Unit, Entebbe, Uganda; Medical Research Council International Statistics and Epidemiology Group, London School of Hygiene and Tropical Medicine, London, UK; University of California San Francisco, San Francisco, CA, USA

**Keywords:** HIV prevention, randomized controlled trials, statistical design

## Abstract

**Objectives:**

Vigorous discussions are ongoing about future efficacy trial designs of candidate human immunodeficiency virus (HIV) prevention interventions. The study design challenges of HIV prevention interventions are considerable given rapid evolution of the prevention landscape and evidence of multiple modalities of highly effective products; future trials will likely be ‘active-controlled’, i.e., not include a placebo arm. Thus, novel design approaches are needed to accurately assess new interventions against these highly effective active controls.

**Methods:**

To discuss active control design challenges and identify solutions, an initial virtual workshop series was hosted and supported by the International AIDS Enterprise (October 2020-March 2021). Subsequent symposia discussions continue to advance these efforts. As the non-inferiority design is an important conceptual reference design for guiding active control trials, we adopt several of its principles in our proposed design approaches.

**Results:**

We discuss six potential study design approaches for formally evaluating absolute prevention efficacy given data from an active-controlled HIV prevention trial including using data from: 1) a registrational cohort, 2) recency assays, 3) an external trial placebo arm, 4) a biomarker of HIV incidence/exposure, 5) an anti-retroviral drug concentration as a mediator of prevention efficacy, and 6) immune biomarkers as a mediator of prevention efficacy.

**Conclusions:**

Our understanding of these proposed novel approaches to future trial designs remains incomplete and there are many future statistical research needs. Yet, each of these approaches, within the context of an active-controlled trial, have the potential to yield reliable evidence of efficacy for future biomedical interventions.

## Introduction

The effectiveness of antiretrovirals (ARVs) as pre-exposure prophylaxis (PrEP) for human immunodeficiency virus (HIV) prevention has improved as we have progressed from pills to longer acting formulations [1–4], and the evidence of treatment as prevention (TasP) as highly effective continues to grow with the success of the 95-95-95 implementation [5–8]. There are continued efforts to further advance HIV prevention. The Antibody Mediation Prevention (AMP) trials of passively-infused VRC01 monoclonal antibody (mAb) provided proof of concept that mAbs can prevent HIV acquisition with viruses susceptible to the specific mAb [9, 10], and combinations of mAbs are being pursued for greater and broader protection [11]. There remains no effective HIV vaccine; the most recent vaccine trials have not found evidence of protection [12–14]. The goal of ongoing HIV prevention research is to ‘expand the toolbox’ of HIV prevention, providing a range of consumer product choices for varied cultural, lifestyle, and healthcare contexts.

Given recent advances in effective prevention, major challenges exist for designing future HIV prevention efficacy trials [15–17]. The core tenets that establish efficacy in placebo-controlled randomized controlled trials (RCTs) are: (1) randomization reliably results in groups that are comparable in both measured and unmeasured characteristics, and (2) high quality conduct results in groups with the same likelihood of exposure and contemporaneous outcome measures during follow-up (i.e., exchangeability). This yields results where the cause of observed differences can be reliably attributed to the randomized intervention. Since oral PrEP (daily tenofovir disoproxil fumarate/emtricitabine (TDF/FTC)) was proven effective for HIV prevention, subsequent ARV-based PrEP trials have been active-controlled, i.e., TDF/FTC was used as the comparator rather than a placebo. Trials of mAbs and vaccines have been placebo-controlled to date, but as highly effective longer-acting products become available, continued use of placebo-control will require careful consideration and justification [18, 19].

Typically, a non-inferiority trial design is used for active-controlled trials. The criterion for ‘success’ for the experimental intervention relies on establishing a narrow difference between the two arms, defined by a pre-specified non-inferiority criterion. The non-inferiority criterion defines what ‘meaningfully inferior’ efficacy is relative to the active control [20]. A common ‘preservation of effect’ approach defines an experimental intervention as non-inferior if it preserves 50 % of the prevention efficacy of the active control (established in historical studies). A second component of the preservation of effect approach promotes using a conservative efficacy estimate for the active control, commonly the lower 95 % confidence limit of the active control from historical trials [21, 22]. A key assumption is that this conservative efficacy estimate holds in the future trial setting (the constancy assumption) [23–25]. It is well-recognized that this is imperfect: there are multiple ways the constancy assumption can be violated [26].

The non-inferiority approach against an active control PrEP product that reduces HIV incidence by 75*−*90 %, can lead to very large trial sizes. Not only is a trial of this size expensive, but the epidemiology of HIV inevitably requires expanding enrollment to populations with lower incidence thereby increasing the risk of not achieving the planned power. In addition, the high prevention efficacy of existing biomedical HIV prevention makes the non-inferiority approach – establishing similar efficacy between experimental and active control – challenging. Given the difficulty of non-inferiority design for HIV prevention, alternative strategies for assessing efficacy of novel prevention interventions are needed.

Establishing “absolute” efficacy, (i.e., efficacy compared to placebo) may be less difficult than establishing non-inferiority for highly efficacious interventions. Given that absolute efficacy is of key interest for guiding regulatory decisions, public policy, and clinical recommendations, we focus on scientifically rigorous pathways for establishing the absolute efficacy of new biologics, e.g., HIV vaccines, and new small (e.g., ARV) or large (e.g., mAb) molecule formulations. Absolute efficacy requires a clearly defined placebo estimand. Where approved biomedical interventions are widely available, it is not immediately clear whether a future target placebo estimand will be standard of prevention without any biomedical intervention (i.e., replacement placebo), or participant choice of available biomedical interventions, or study-provided access to specific biomedical interventions. The latter choices are appropriate if the new intervention has potential for use in conjunction with currently approved agents, and conceptually, the placebo replaced only the new intervention. In this case, interest is efficacy of the new intervention in the context of “real-world” use of approved agents as part of the standard of prevention. Our focus in this article, however, is the “replacement placebo” estimand, where we estimate effectiveness of the experimental compared to no biomedical intervention. We use the term “counterfactual placebo” throughout to convey the intended replacement placebo estimand, as if, counter-to-fact, the future trial had a placebo control to estimate absolute efficacy in the context of best (non-biomedical) standard of prevention. The purpose of this choice of language is to indicate the intent to closely mimic a placebo-based estimand of absolute efficacy.

To discuss active control design challenges and identify solutions, a virtual workshop series was hosted and supported by the International AIDS Enterprise between October 2020 and March 2021 [27]. Ongoing subsequent symposia discussions continue to advance these efforts [28, 29]. This paper summarizes approaches under discussion, including a description of the study design, examples of how it has been used, key assumptions, and strengths and challenges of the approaches in the context of available, effective HIV prevention drugs with efficacy of 75–90 %.

**Figure 1:**
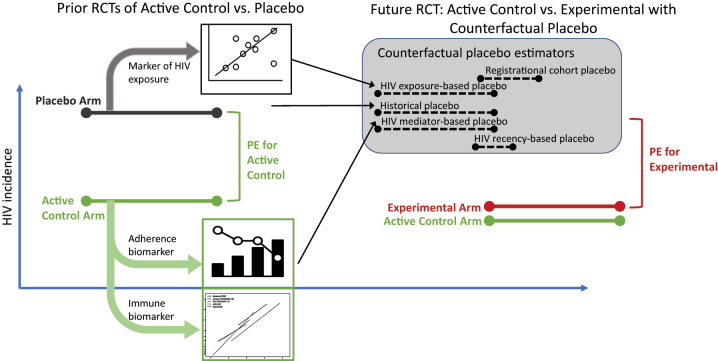
Multiple approaches for inferring prevention efficacy (PE) of an experimental intervention relative to placebo, based on data from an active-controlled trial.

Below we describe and discuss six potential study design approaches for formally evaluating absolute prevention efficacy given data from an active-controlled HIV prevention trial (summarized in Figure 1 and Table 1). Of note, we adopt three important principles from non-inferiority trial design in our proposed alternative design approaches: (1) randomization of experimental intervention vs. active control; (2) efficacy of the experimental intervention is not substantially lower than the active control; and (3) non-randomized comparisons (e.g., historical data) need to have safeguards that account for measurement error and risk of bias from potential confounding.

**Table 1:** Conceptual approaches to inferring prevention efficacy of an HIV prevention intervention relative to a counterfactual placebo.

Concept	Key assumptions	Candidate setting	Strengths	Weaknesses
**Approaches that infer counterfactual placebo HIV incidence**				
*1. Registrational cohort* A registrational cohort, or trial ‘run-in’, is enrolled and followed for incident HIV	– Constancy of HIV incidence over time for registrational and trial f/u – Balanced cohort vulnerability factors between registrational and active-controlled trial	Delay in trial initiation and no/limited access to active control agent during registration cohort	– Quality capture of incident HIV during both time-periods – Assessment in same population and settings – Consistency in covariate measurement	– Participants in the registrational cohort may use PrEP through local access reducing incidence relative to placebo – Maintaining equivalent inclusion criteria – Potential bias from censoring – Limited control of the registration cohort duration
*2. Recency assays during trial* s*creening* HIV recency assays, applied to individuals screened for active-controlled trial participation	– Constancy of HIV incidence over time of recency assay period plus trial f/u – Balanced cohort vulnerability factors between recency sample and active-controlled trial	Screening large number of individuals not currently on biomedical prevention for active-controlled trial participation	– HIV incidence assessment is time adjacent – Assessment in same population – Consistency in covariate measurement	– Ensuring the screened cohort includes individuals with recent HIV acquisition from the target population – Statistical precision – Required constancy assumption
*3. External trial placebo arm data* Placebo arm data for historical or concurrent placebo-controlled HIV prevention trials	– Constancy of HIV incidence over time for historical trial f/u and active-control trial f/u – Ability to achieve similar vulnerability factors between historical placebo and active-controlled trial	Concurrent or historical placebo-controlled trial(s) conducted in same target population as active-controlled trial	– Known placebo data prior to trial – Use of individual-level data from clinical trial participants – Quality capture of incident HIV during both time-periods	– Potential for significant time to elapse between studies – Sites may differ
*4. Biomarker of HIV exposure* HIV incidence/exposure biomarker association estimated using historical placebo cohort data. Exposure biomarker measured in active-control trial and used to predict HIV incidence	– Invariant exposure biomarker, not modified by active control and/or experimental intervention – Association between HIV and exposure biomarker consistent across external cohorts and active-controlled trial	Biomarker a strong predictor of HIV, e.g., non-HIV STI	– Known biomarker data prior to trial	– Requires highly correlated biomarker in multiple external placebo cohorts – Precision of the counterfactual placebo HIV incidence estimate depend on the number of external placebo cohorts
**Approaches that infer prevention efficacy of experimental intervention**				
*5. ARV drug concentrations as mediators of ARV PE* Drug concentration of ARV is established as mediator of PE, and PE is estimated leveraging mediation model based on historical placebo-controlled ARV RCT(s)	– Drug concentration is a mediator of ARV PE – No missing modifiers of PE	ARV is active-control arm. Placebo-controlled historical trials of ARV conducted with same drug concentration assay as in the active-controlled trial	– Monotonic dose-response relationship between adherence biomarker and PE observed to date for ARV-based prevention	– Need least one placebo-controlled efficacy trial of the active control demonstrating evidence that an adherence marker is a mediator of PE – Limited knowledge of adherence-PE relationship
*6. Immune biomarkers as mediators of mAb/vaccine PE* Immune biomarker established as mediator of protection in prior placebo-controlled trial(s). Immune biomarker is measured for mAb/vaccine arm in active-controlled trial, and used to estimate PE	– Immune biomarker is a mediator of mAb/vaccine PE – No missing modifiers of PE	Vaccine or mAb as active-control arm. Placebo-controlled historical trial(s) where vaccine/mAb is efficacious and same immune biomarker as in the active-controlled trial	– Proven pathway to subsequent regulatory approval for vaccines – Well-developed statistical methodology	– At least one placebo-controlled efficacy trial of the mAb/vaccine needs to have been conducted demonstrating immune marker is a mediator of PE – Validity of immune biomarker for future mAb/vaccines is unknown

ART, anti-retroviral therapy; mAb, monoclonal antibody; RCT, randomized controlled trial; f/u, follow-up; STI, sexually transmitted infection; PE, prevention efficacy.

## Registrational cohort

### Concept

Persons eligible for a planned active-controlled RCT are enrolled in a registrational cohort with access to available standard of care for HIV prevention and regular HIV testing and follow-up. Operationally, the registrational cohort is put in place during the planning period before regulatory approval to begin enrollment in the RCT. When the RCT opens to enrollment, those in the cohort (who did not acquire HIV) are re-screened for participation, as well as newly recruited subjects, and are randomized to active control or experimental intervention. The observed incidence in the registrational cohort provides a ‘counterfactual placebo’ HIV incidence rate estimate. Absolute efficacy of the experimental product can be estimated as a contrast of the incidence in the experimental arm compared to the registrational cohort.

### Example

The ongoing PrEPVacc study (ClinicalTrials.gov #NCT04066881) is a leading example of the registrational cohort approach [30]. While start-up activities (e.g., approvals, database preparation, training, etc.) for PrEPVacc were underway, eligible participants could enroll into a registrational cohort and were followed until PrEPVacc opened for recruitment. During this time, cohort participants received HIV testing every 3 months and the standard of care for HIV prevention including linkage to a local PrEP provider. At the opening of the randomized trial, registrational cohort participants were re-screened for eligibility and randomized to one of three study arms. Absolute efficacy of the PrEP regimens will be evaluated using the averted infection ratio [31], a measure that contrasts newly diagnosed HIV for PrEP regimes in the context of counterfactual placebo incidence informed by the registrational cohort.

### Assumptions, strengths, and weaknesses

A key assumption is that HIV incidence is constant across registrational and trial follow-up time points. A strength of this design is high quality capture of incident HIV in both registrational cohort and active-controlled trial periods, and the potential for constancy in HIV exposure across the registrational and active control trial cohorts because of substantial cross-over from the registrational cohort to the RCT, which ensures similarity of populations. Consistency in covariate measurement across the registrational cohort and trial follow-up periods allows baseline and time-varying covariate adjustment methods, which rely on the assumption of constancy of HIV incidence conditional on measured covariates. Additionally, if the duration of the registrational cohort follow-up allows a temporal trend in HIV incidence to be estimated and extrapolated from the registrational cohort follow-up period into the RCT follow-up period, the assumption of time-constancy in HIV incidence may be relaxed. A formal statistical formulation for the placebo based on registrational cohort is lacking; we note that this trial design is based on the novel averted infection ratio estimand [32].

A challenge with this approach is while some of the registrational cohort may have “placebo” risk (i.e., during the ‘efficacy-implementation’ gap between when an intervention has been found effective and when it is available to individuals behaviorally vulnerable to HIV) [33, 34], increasingly participants in the registrational cohort may uptake PrEP, leading to reduced cohort incidence relative to “placebo”. However, as this potentially underestimates counterfactual placebo incidence, relative efficacy estimates for the experimental relative to the counterfactual placebo of the registrational cohort would likely be conservative. Other challenges with the registrational cohort approach are maintaining equivalent inclusion criteria and screening procedures in both phases, potential bias from censoring due to HIV acquisition before randomization, and possibly limited control of the duration (therefore statistical information accumulated) of the registrational cohort phase.

## Recency assays at trial screening

### Concept

Recent infection testing algorithms (RITAs) are used to estimate counterfactual placebo incidence among participants screened for the active control RCT. RITAs are a pre-specified combination of laboratory assays developed to classify HIV acquisition as test-recent or not [35]. RITAs were developed and validated using stored samples from persons with a known time interval when HIV acquisition occurred, establishing for each RITA, the average duration that an individual continues to test recent after new HIV diagnosis (“window period”) [36–39]. HIV incidence in the recent past is then estimated based on the proportion of test-recent individuals encountered during screening for the RCT, leveraging the relationship between prevalence and incidence [40]. This estimate serves as a counterfactual HIV placebo incidence rate. Eligible individuals without HIV are randomized to experimental or active control arms, and HIV incidence based on recency assays is contrasted with experimental arm incidence during trial follow-up.

The statistical framework for this design is defined in Gao et al. [41] In addition, building on methodological work of others [42–44], sample size requirements for a hypothetical trial using current RITA-based incidence estimates for an experimental intervention trial have been defined as a function of the number of screened individuals in settings similar to recent HIV prevention trials in men who have sex with men (MSM) and African cisgender women [42–44].

### Examples

Two ongoing active-controlled Phase 3 efficacy trials evaluating new PrEP agents are using recency testing to estimate counterfactual placebo rates (ClinicalTrials.gov #NCT04994509 and #NCT04925752) [45, 46]. In PURPOSE1, young women behaviorally vulnerable to HIV, and in PURPOSE2, MSM, are randomized to Lenacapavir (the experimental agent) or the active control (FTC/TDF) for PrEP. An incidence phase of each trial will use cross-sectional recency testing to establish the background, or counterfactual, rate of HIV acquisition, which will contribute to primary analysis establishing the prevention efficacy of Lenacapavir.

### Assumptions, strengths, and weaknesses

A naive approach based on comparing HIV incidence among those screened with incidence during trial follow-up relies on the key assumption of constancy in HIV incidence over the RITA ‘window period’ and trial follow-up periods. Conditioning on covariates measured at screening can reduce risk of confounding, although there are likely unmeasured differences between individuals screened vs. enrolled in a trial. The assumption of constant incidence over time may be violated by secular trends in HIV incidence. Methodological advances are needed to accommodate these potential sources of bias.

A strength of this approach is, as with the registrational cohort approach, HIV incidence estimates from the screening and follow-up periods are based on the same sites participating in the trial, and specific behavioral and demographic characteristics can be measured among all eligible screenees. In addition, incidence based on recency assays conducted throughout the enrollent period has some temporal overlap with the trial follow-up period. Enrolled participants who are without HIV at screening contribute to both the recency-assay based incidence and on-trial longitudinal evaluation of HIV incidence; this cross-over is substantial given the relatively low incidence of HIV and ensures similarity between the two groups. 

A practical challenge with the approach is ensuring the screened cohort includes individuals with recent HIV acquisition from the target population, which is required to ensure an unbiased estimate of HIV incidence for this target population. To achieve this, trial screening must not exclude persons known to be newly diagnosed, meaning first tested positive within the prior two years (the currently defined window limit of recency assays). This may be difficult to achieve in communities with frequent HIV testing, as persons who know they have acquired HIV are likely to be on ART (potentially affecting recency assay outcomes) and less likely to screen for an HIV prevention trial. Strategies to ensure unbiased representation from recently infected persons are important for this approach.

While previous work suggests that adequate power can be achieved with reasonable numbers screened, in general statistical precision is a concern for this approach [41]. A large number of screenees are needed to yield sufficient test-recent samples for adequate precision of the counterfactual placebo incidence estimate. Further exacerbating the issue, persons screening for the trial who are currently using PrEP, and thus not at placebo risk, may need to be excluded from the trial or the RITA-based-incidence estimation. In settings where regular HIV testing is routine, precision may be increased by utilizing information about previous HIV-negative and positive tests. Additional precision may be gained with use of continuous, rather than binary, RITA outcomes (i.e., monotonic change over time since acquisition, rather than recent/not recent). Methodology advancement is needed on these fronts.

## Placebo HIV incidence from external trials

### Concept

Use of individual-level HIV and covariate data from placebo arms of historical or contemporary external trials to estimate placebo HIV incidence for an active-controlled trial population. This is an example of using external data as a control for which there is a large methodological literature [47–50]. Regulatory guidance from the US Food and Drug Association (FDA) about use of external controls was updated in 2019 and notes the advantages of external individual-level data from other clinical trials in similar populations [51, 52]. In HIV prevention, it is common for trial sites to participate in multiple RCTs evaluating different interventions. Therefore, application of this approach may have the advantage of adequate power using only clinical trial sites common between the two trials. If the epidemiologic characteristics of the HIV epidemic in the target population remain broadly similar, it is credible to assume that rates of HIV incidence observed in prior placebo arms at the site are a reliable estimate of counterfactual placebo incidence.

### Examples

This approach was initially illustrated in HIV prevention studies with sero-different couples, a setting where the exposure likelihood from a partner with HIV was known and plausibly constant across populations and over time [53]. More recently, placebo arm data for HIV vaccine and mAb efficacy trials conducted during 2015–2021 supplied external data for estimating counterfactual placebo efficacy of twice-monthly injectable cabotegravir (CAB-LA) compared to FTC/TDF in HPTN 083 (ClinicalTrials.gov #NCT02720094) and HPTN 084 (ClinicalTrials.gov #NCT03164564), both active arm Phase 3 trials [54]. Importantly, the placebo-controlled vaccine and mAb trials were simultaneously enrolling in similar regions at partially overlapping sets of clinical trial sites, with similar eligibility criteria.

In related work, data from the placebo-controlled efficacy trial of the Dapivirine vaginal ring (ASPIRE: ClinicalTrials.gov #NCT01617096) [55] were used to estimate effectiveness of the ring in the subsequent open-label extension study (HOPE: ClinicalTrials.gov #NCT02858037) [56]. Importantly, because the trial had a placebo-controlled randomized and blinded phase, uniformity in capture of incident HIV and behavioral factors between the blinded and open-label study periods, as well as the considerable overlap between participants in ASPIRE and HOPE, this comparison required weaker assumptions for validity. To date, these examples reflect estimates of prevention efficacy in various observational settings, not in support of primary efficacy estimates.

### Assumptions, strengths, and weaknesses

The key assumption underlying this approach is constancy of counterfactual placebo HIV incidence between the external study placebo arms and the current trial, conditional on covariates. As time passes, changes in the epidemiology of HIV will likely render the validity of historical comparisons increasingly tenuous. Unmeasured differences in behavioral vulnerability to HIV between participants in external studies and the current study are a threat to validity. And for HIV behavioral vulnerability factors that are measured, questionnaires in distinct trials are rarely identical. For example, in a recent counterfactual comparison across four studies [54], the only covariates collected the same way in all studies were age and baseline sexually transmitted infection (STI).

A strength of this approach is use of individual-level data from clinical trial participants: both external trials with placebo arms and active-controlled trials assess newly diagnosed HIV through prospective HIV testing, are conducted in populations receiving a high standard of prevention and care, and maintain high retention and quality covariate capture. Of note, a simple and applicable statistical framework is to use a direct standardization approach to construct a counterfactual placebo arm with matching distribution of a select set of covariates, e.g., race and country, HIV risk score [57]. Propensity scores and more sophisticated causal inference approaches based on a potential outcomes framework are also statistical methods appropriate for this setting [47, 58, 59].

Given advances in HIV prevention, we expect few future placebo-controlled HIV prevention efficacy trials without concomitant use of biomedical prevention. If conducted, placebo-controlled trials might be within highly selected subpopulations not eligible or willing to use existing preventive interventions [60] that would not generalize to broader populations eligible for active-controlled trials. Alternatively, placebo-controlled trials might be ‘layered’ on top of a highly effective standard of HIV prevention [15], thus providing estimates of placebo incidence in the context of extant use of additional biomedical prevention. Statistical methodology to estimate the “counterfactual placebo” rate of acquisition without biomedical prevention in contexts where a proportion of the external cohort was using biomedical prevention (e.g., FTC/TDF, CAB-LA) is needed.

## Biomarkers of HIV exposure (e.g., sexually transmitted infections (STIs))

### Concept

A reliable biomarker of exposure to HIV would permit a conclusion that a very low rate of HIV acquisition in an active-controlled trial was a result of protection by an intervention. For example, if incidence of other STIs, such as syphilis and gonorrhea, were high and similar across arms in an active-controlled trial, we infer ongoing sexual contact with partners with STIs. As markers of ongoing sexual contact, these might serve as biomarkers of HIV exposure.

### Examples

The approach was suggested in the DISCOVER trial (ClinicalTrials.gov #NCT02842086), an active-controlled trial comparing two oral PrEP regimens, and HPTN 083, an active-control trial comparing oral and injectable PrEP, where high STI rates were noted in both arms of these studies while low HIV incidence was observed [3, 61]. Inferring counterfactual placebo HIV incidence from rectal gonorrhea (RGC) has been proposed for the MSM population [16, 62–64]. A body of cohort studies without biomedical HIV prevention models the association between cohort-level incidence of the STI and HIV i.e., the association under a counterfactual placebo. The observed STI incidence rate in an active-controlled trial, under this model, could then be used to estimate counterfactual placebo HIV incidence. The concept received support from the FDA advisory committees, and the FDA itself endorsed the approach in guidance to industry [65].

### Assumptions, strengths, and weaknesses

As articulated in Zhu et al. [64] the approach relies on three key assumptions. First, it requires that the correlation between HIV incidence and STI incidence applies across external cohorts and the active-controlled trial. This assumption is challenging to validate given the many biological, behavioral, and epidemiological factors that influence population incidence of STIs. In particular, it is possible that scale up of HIV treatment and prevention could reduce HIV incidence but not affect incidence of other STIs, thus modifying the HIV–STI correlation. The second key assumption is that the interventions evaluated in the active-controlled trial do not change STI incidence. It is not possible to fully validate this assumption. There is data supporting the absence of an effect of ARV-based prevention on multiple STIs [2, 3, 5], but this does not necessarily apply to a future intervention. The third assumption is that the association between HIV and STIs can be estimated given the external cohort data.

Application of the approach requires multiple external cohorts to estimate the HIV–STI correlation. Precision of the resultant counterfactual placebo HIV incidence estimate depends on the number of external cohorts. This is a major practical limitation of the approach, and the aforementioned point about the impact of successful HIV prevention on the HIV–STI correlation suggests that useful cohorts are likely to be historical cohorts before the advent and rollout of ARV-based HIV prevention.

The current statistical framework relies on study-level data for the external cohorts. Extension of this framework to allow individual-level data is warranted and would allow for more precise estimation and adjustment for measured covariates that potentially influence both HIV and STI incidence, thus allowing for weaker assumptions for validity.

## Adherence biomarker as a mediator of prevention efficacy for ARVs

### Concept

Use an adherence biomarker as a mediator of prevention efficacy of the active control in the active-controlled trial setting [66]. With an estimate of active control prevention efficacy in hand, the absolute efficacy of the experimental intervention, relative to placebo, can be estimated. Combining the estimated active control prevention efficacy with the HIV incidence of the experimental arm leads to an estimate of the counterfactual placebo HIV incidence.

### Example

The DISCOVER trial in which oral FTC/TDF served as the active control for emtricitabine plus tenofovir alafenamide (F/TAF), measured adherence to FTC/TDF using dried blood spots [66, 67]. The estimation of prevention efficacy of FTC/TDF was based on the adherence-efficacy association for oral FTC/TDF, using prior studies; this was then used to estimate efficacy of F/TAF relative to a counterfactual placebo.

### Assumptions, strengths, and weaknesses

The key assumptions of this approach are: (1) the adherence biomarker accurately estimates the active control prevention efficacy; and (2) the adherence-efficacy dose-response association holds across the trials (historical and current active-controlled). Importantly, the adherence biomarker need not fully explain efficacy; additional factors predicting efficacy can be included in the estimation. Glidden and colleagues formulated a Bayesian approach to estimation that imports the uncertainty in the biomarker/efficacy relationship from previous trials and propagates them into the active-controlled trial [66]. A feature of the Bayes framework is the ability to incorporate multiple sources of data (e.g., proportion screened in the eclipse phase of HIV infection and historical infection rates) to inform the Bayesian prior for the model parameters.

To apply this approach, one needs at least one placebo-controlled efficacy trial of the active control completed, with strong evidence established that an adherence marker is a mediator of prevention efficacy. The transportability of the adherence-efficacy association needs to be credible, as this is the basis for the assumption that this association holds for the current active-controlled trial. As well, the same adherence marker must be measured in the historical trial(s) and the current active-controlled trial.

The assumption that the adherence-efficacy association for the active control transports to the current active-controlled trial is similar to the ‘constancy’ assumption of a non-inferiority formulation. However, whereas under a non-inferiority framework it is assumed that the efficacy of the active control transports to the new setting, the assumption here is weaker: it is that the efficacy given a measured level of an adherence biomarker transports to the new setting. Sensitivity analyses to evaluate how conclusions might change under violations of constancy are important.

The approach depends on a monotonic dose-response relationship between the adherence biomarker and prevention efficacy. Methodology developed has a highly parametric formulation – more flexible estimation would be desirable. A small number of HIV acquisitions observed in the active control arm will result in a highly uncertain estimate of the preventive efficacy and placebo incidence. An open research question around this design is an approach to determining sample size, with consideration to adaptation to emerging data on adherence.

## Immune biomarker data as a mediator of prevention efficacy for mAbs and vaccines

### Concept

Use of an immunological marker demonstrated to mediate efficacy of the intervention, where mediation is formalized using the natural direct and indirect effects framework of causal inference. This concept is derived from previous immunological assays that measure actively or passively generated immune responses following vaccination or dosing as useful predictors of prevention efficacy and accepted surrogate endpoints for approving vaccine regimens [68–70] including the HIV vaccine research field, which has developed vaccine-induced immune responses as valid surrogate endpoints (i.e., reliable predictors of vaccine efficacy) [71, 72]. The approach is conceptually similar to approach 5 (adherence biomarker as a mediator of prevention efficacy for ARVs) for ARV-based active control interventions, where the immunological biomarker substitutes for the adherence biomarker used in the ARV setting.

### Examples

In the AMP trials (ClinicalTrials.gov # NCT02568215 and # NCT02716675) evaluating the prevention efficacy of the VRC01 mAb for HIV prevention [9], Gilbert et al. [73] generated evidence that the predicted serum neutralization 80% inhibitory dilution titer (PT80) can predict the level of prevention efficacy of VRC01, and likely, other mAb regimens. PT80 is a biomarker that quantifies the neutralization potency of antibodies in an individual’s serum against an HIV-1 isolate. Should this biomarker be validated as a predictor of prevention efficacy in another trial of a mAb regimen [10], it may be possible to use PT80 as a surrogate endpoint for basing approval decisions of future mAb regimens that target the same epitopes.

The RV144 ‘Thai Trial’ (ClinicalTrials.gov # NCT00223080) evaluating a clade A/E pox-protein-based HIV vaccine, and subsequent laboratory and pre-clinical follow-up studies provided the first reliable evidence that a set of vaccine-induced immune responses–Fc-mediated antibody responses directed at the V1V2 region of the HIV envelope, and polyfunctional CD4+ cellular immune responses correlate inversely with HIV-1 acquisition [72, 74]. Results of the immune correlates analyses of the Adenovirus virus serotype 26 (Ad26)-vectored mosaic vaccine in HVTN 705 (ClinicalTrials.gov #NCT03060629) [75] also support a potential role for these immune responses in mediating the efficacy of HIV vaccines. These or other immunological biomarkers, if validated in future HIV vaccine trials, may potentially be viable predictors of vaccine efficacy [75, 76].

### Assumptions, strengths, and weaknesses

Like the adherence biomarker mediation approach, the key assumptions of this approach are that active control prevention efficacy is mediated through the immunological biomarker, and that the biomarker-efficacy association holds across historical trials and the current active-controlled trial. For use, at least one placebo-controlled efficacy trial of the active control needs to have been conducted, with strong evidence establishing that the biomarker is a mediator of prevention efficacy. The transportability of the biomarker-efficacy association to other mAb or vaccine mechanisms needs to be evaluated, as this is crucial for validating the assumption that this association holds for future active-controlled trial. Standardization of the immunological biomarker measurement is key, across the historical trial(s) and current active-controlled trial, including specimen type, timing of specimen collection relative to intervention, and immunological assay and laboratory.

## Discussion

Judged against the gold standard of a placebo controlled RCT, the proposed approaches require assumptions to ensure validity of the efficacy estimate. A well-designed non-inferiority trial, with a predefined non-inferiority margin based on prior placebo controlled RCTs of an effective intervention, is an accepted method for establishing efficacy of an experimental intervention. (Although for vaccines, clinical endpoint non-inferiority trials have rarely been conducted; instead, the dominant approach for subsequent vaccine approval has been through validated or partially validated immunological marker surrogate endpoints.) The non-inferiority trial assumes constancy of active control efficacy between settings, and generally accepted guidelines set a high bar for establishing non-inferiority, to ensure high confidence the experimental intervention is effective. As we consider novel approaches for establishing efficacy for new interventions through active-controlled trials, it is important to build similar safeguards in these trial designs to mitigate the lack of randomized comparison against a placebo arm. “Success criteria” for these novel design approaches needs to be pre-specified, and explicit allowances made for potential violation of assumptions and statistical uncertainty in external estimates.

One important design safeguard that we recommend retaining, as in non-inferiority designs, is inclusion of an established biomedical intervention as a randomized active control. Even if the primary comparison is between the experimental intervention and a counterfactual placebo, and the contrast between experimental intervention and active control is not necessarily fully powered, this provides an important safeguard against an ineffective intervention: against a 75*−*90 % effective active control, an ineffective drug would be apparent. This also preserves the careful ethical framework of the non-inferiority trial, with a substantial proportion of participants assigned to known effective prevention. Informative direct comparison of the safety profile of the two biomedical interventions is available. Lastly, it permits leverage of historical placebo-controlled data on efficacy of the active control, based on direct assessment of incidence and/or efficacy in the active control arm.

Each of the proposed approaches makes key assumptions of constancy between measurements despite not being taken at the same time and/or for the same cohort. Indeed, the general framework for the assumptions of constancy is the gold standard established by high quality, contemporaneous follow-up of a placebo controlled RCT cohort. Many of the proposed approaches leverage data from completed (or contemporaneous) clinical trials, which have the advantage of individual-level data with well-characterized eligibility criteria, a wealth of measured covariates, high quality retention with a consistently high standard of prevention and care, and a laboratory-based assessment of HIV incidence. Development of a typology of comparability, which describes distance in elapsed time and compares by-group cohort characteristics relative to the “RCT ideal”, as part of a proposed counterfactual placebo estimand, would be a useful qualifier for the quality and reliability of the evidence for prevention efficacy. Pre-defined success criteria that build in appropriate conservatism to account for assumptions of constancy are also necessary to result in trials that provide a reliable judgement about efficacy of future experimental interventions. While we discussed each of the approaches separately to articulate their merits and differences, in practice, trials are likely to leverage multiple data sources and approaches to estimate absolute efficacy of a novel intervention tested in an active control design. Future work on potential merits and approaches for combining evidence across approaches would be valuable.

For observed differences in measured characteristics between cohorts, statistical adjustment methods are well developed and characterized. These can be used to predict effects or incidence at the value of observed cohort means. Careful planning to ensure individual vulnerability factors and potential modifiers of the biomarker laboratory assays are consistent across trials is an important feature for future success of these novel approaches. For example, the US government’s COVID-19 Vaccine Correlate of Protection Program prioritized use of the same neutralizing antibody marker across studies [72], resulting in successfully characterization of a biomarker of efficacy.

An ongoing challenge for the counterfactual estimate of placebo incidence (i.e., with no biomedical intervention) is increasing use of biomedical prevention in high incidence populations. Future trials using cross-sectional incidence will have to contend with screening from a population with access to HIV treatment and available forms of PrEP. A trial could screen only persons not currently using PrEP – the approach used for the HVTN 706 (ClinicalTrials.gov #NCT03964415) and PURPOSE trials. We note the potential complications arising from prior use of long-acting PrEP, as the active drugs can remain detectable, and possibly partially effective, for many months. Alternatively, counterfactual incidence could be assessed relative to a context of current level of PrEP use, rather than of absolute efficacy similar to the mAb trials.

The complementary challenge for trials that use historical placebo data is ongoing evolution of the epidemic, especially accounting for the impact of increasingly available and highly effective HIV prevention. While statistical methods can potentially update likelihood based on individual participant characteristics (i.e., age, PrEP use), credibly “bridging” HIV acquisition rates to update the historical data for evolution of the epidemic remains difficult. A current illustration is the decrease in HIV incidence credited to increased viral suppression because of ARV treatment.

## Future statistical research

As we face the reality of a prevention field with great successes achieved, but with vaccines and other important potential products still needing to advance, our understanding of these proposed novel approaches to future trial designs remains incomplete. Needs for future statistical research are to:

– Articulate a framework for pre-defined success criteria appropriately conservative under violation of assumptions.
– Study the performance of strategies to mitigate the risk of imprecision in the counterfactual placebo-based estimates.
– Evaluate mediation of specific adherence/pharmacokinetic measures for ARV-based interventions; challenge is potentially different loci of activation for different drug formulations or classes.
– Evaluate mediation of immune biomarkers for mAbs; challenge is limited inter-individual variability.
– Develop methods for combining sources of information, and/or formally combining prevention efficacy estimates. Data fusion and Bayesian frameworks could be utilized.
– Advance methods for covariate adjustment, where not available, e.g., leveraging recency assays.
– Establish a common set of statistical simulation scenarios to facilitate sensitivity analysis, e.g., defining a range of evaluation settings in which assumptions are satisfied and violated.
– Advance methods that characterize both statistical uncertainty and uncertainty due to unidentifiable parameters.
– Develop and test sample size considerations for each proposed approach.

Since the completion of the original workshop series CAB-LA has been licensed for HIV prevention with an observed HIV incidence of *<*0.5*/*100 person-years [2, 3]. HIV prevention research is thus advancing in a context of multiple, highly effective biomedical prevention products. Somewhat parallel dilemmas unfolded with the successes achieved in COVID-19 vaccines. Following multiple placebo-controlled RCTs of novel COVID-19 vaccines that established efficacy, new vaccines were licensed based on surrogate endpoints/immune correlates, trials of new vaccines proceeded with continued use of placebo, and proposals were developed to use non-inferiority trials with novel justifications for establishing absolute efficacy against an active control [73, 77]. In other fields that have achieved prevention success we observe a shift to non-RCT methods for establishing comparative efficacy [78].

The approaches we describe for evaluating the efficacy of new interventions in HIV prevention are being implemented in current clinical trials. To meet the imperative of developing new prevention drugs, and to advance vaccines, trial design approaches that retain evaluation rigor, together with a thorough understanding and assessment of their statistical properties, are needed. Each of these approaches, within the context of an active-controlled trial, has the potential to yield reliable evidence of efficacy for future biomedical interventions.
